# A monitoring and evaluation gap for WHO’s community health worker guidelines, Botswana

**DOI:** 10.2471/BLT.19.243238

**Published:** 2020-03-26

**Authors:** Stephanie Watson-Grant, Ratanang Balisi, Deborah Kaliel, Styn Jamu, James Thomas

**Affiliations:** a MEASURE Evaluation, Carolina Population Center, University of North Carolina at Chapel Hill, 123 W Franklin Street, Chapel Hill, NC, 27516, United States of America (USA).; b USAID Mission, Gaborone, Botswana.; c USAID Office of HIV/AIDS, Washington, USA.; d P/Bag 00421, Plot 21606, Phakalane, Gaborone, Botswana.

Since the Alma-Alta Declaration in 1978,^1^ community health workers (CHWs) have been recognized as an important component of primary care. A World Health Organization (WHO) review^2^ found 71 publications providing evidence for the effectiveness of CHWs in areas such as maternal and child health, infectious diseases and noncommunicable diseases. The evidence came from publications and stakeholder interviews in both high and low- and middle-income countries in sub-Saharan Africa, South Asia and the Americas. Weak or no evidence for the effectiveness of CHWs was found only for diabetes and cancer care in low- and middle-income countries and for malaria in high-income countries.

Although CHWs are known to be effective, many nations that signed the Alma-Alta Declaration have yet to include CHWs in their human resources for health policies or to develop systems for monitoring and evaluation of CHWs’ performance. In the WHO Region of the Americas, gaps have been identified in health workforce policy and in human resources for health monitoring and evaluation systems.^3^ The European Health Information Gateway provides detailed information on human and technical resources for health in the WHO European Region Member States.^4^ However, Health 2020,^5^ the overarching European health policy, mentions the health workforce only in the context of training. In the WHO Western Pacific Region, 10 of the 14 countries have a health policy or plan for human resources, two specifically mention CHWs in the policy and eight have systems for collecting relevant data. Although 10 countries in the WHO South-East Asia Region have human resources for health strategies and have adopted a set of indicators across the countries,^6^ there is no mention of CHWs in the policies or indicators. We found no evidence of human resources for health policies in the WHO Eastern Mediterranean Region. Seventeen of the 47 countries of the WHO African Region have a health policy for human resources and four have a relevant information system, but we found no mention of CHWs in the policies.^7^

In October 2018, WHO launched guidelines^2^ on health policy and system support to optimize the role of CHWs. The document outlines 15 recommendations for improving CHW programmes which emerged from a systematic review of over 122 reviews and interviews with 96 respondents.^8^ The recommendations can be categorized as relating to selection and training (5 recommendations), terms of service (4 recommendations) and programme implementation (6 recommendations). 

Two years earlier, in 2016, the Botswana government had launched their Treat All strategy for human immunodeficiency virus (HIV) infection. This policy followed from WHO’s HIV treatment guidelines of 2015^9^ advising that anyone infected with HIV should start receiving antiretroviral treatment (ART) as soon as possible. Among the barriers the country faced was the capacity of facilities to increase their reach and yields of testing to ensure linkages to treatment. One solution identified was to share rapid HIV testing roles between facilities and CHWs. The Botswana health ministry therefore commissioned an analysis to examine how to share facility-based tasks with CHWs to advance the Treat All policy. We describe here the analytical process, using ART medication refills as an example, and discuss how the findings compare with the WHO’s CHW guidelines. 

The analysis in Botswana was conducted by MEASURE Evaluation, the United States Agency for International Development (USAID) flagship project for strengthening health information systems. The MEASURE Evaluation team met with 165 stakeholders, individually and in groups. Interviewers collected data through in-depth interviews with senior government officials, United Nations agencies, nongovernment organizations, donor communities, civil societies and health association leadership. Interviewers also gathered data in group discussions with district health management teams, donor communities (USAID and the United States Centers for Disease Control and Prevention), community members and CHWs. 

The analysis was structured by combining USAID’s Local Systems Framework^10^ with causal loop diagramming, a systems analysis tool. The framework names five key components of local systems, known as the 5Rs (rules, roles, relationships, resources and results). The information gathered at the interviews and stakeholder meetings were organized using the 5Rs framework and then graphically presented as causal loop diagrams. These diagrams^11^ are analytical tools based on principles of system dynamics to analyse and understand how systems behave, interact with their environment and influence each other.^12^

Six tasks were discussed at the meetings: (i) HIV testing; (ii) ART medication refills; (iii) contraceptive pill refills; (iv) family planning counselling; (v) sexually transmitted infection counselling; and (iv) sputum collection and transportation. Of note, HIV testing in Botswana was already being delivered by some CHWs. Participants during the discussions wanted HIV testing expanded to be delivered by all CHWs. The other five tasks would be newly delivered by CHWs. The MEASURE Evaluation team developed separate causal loop diagrams for each task discussed. The roles, rules and resources and implications of the 5Rs formed the variables (or nodes), which were used to map out the causal relationships for each of the tasks and the relationships as directional connections between the nodes of the diagram. The result of each task was the health outcome agreed by the stakeholders for each task. Once the causal loop diagrams were drafted, leverage points, points where change could produce big shifts, were identified.

**Fig. 1:**
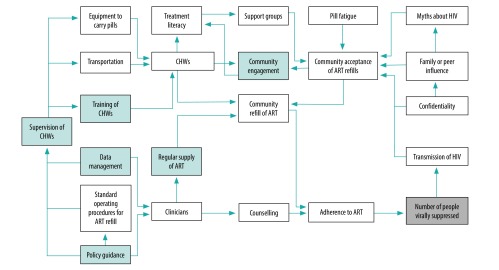
Causal loop diagram of roles, rules and resources of community health workers to change HIV viral load suppression in infected people, Botswana

ART: antiretroviral treatment; CHW: community health worker; HIV: human immunodeficiency virus.

Note: The shaded areas represent leverage points.

Fig. 1. shows the causal loop diagram for one of the six tasks: ART medication refills. The number of people virally supressed is the desired result. An analysis of the leverage points in the causal loop diagram (shaded areas in Fig. 1) generated six recommendations. The first recommendation was to develop policy guidance to harmonize CHW entry qualifications, training, remuneration and reporting structures. Second, guidelines needed to be developed for supervision of CHWs at community, district and national levels. The third recommendation was to develop CHW training activities, such as curricula for use with organizations deploying CHWs and standard operating procedures. Fourth, a monitoring and evaluation framework needed to be developed to address issues of data collection, data analysis and reporting systems for community delivery of HIV services. The fifth recommendation was to assist the central medical stores of the Botswana health ministry to strengthen their supply chain management to accommodate community-based medication refills and supplies. Finally, the analysis identified a need to enhance community acceptance of the new CHW services through a community engagement strategy.

The Botswana analysis was conducted before the publication of WHO’s guidelines on CHWs, yet it arrived at many of the same conclusions. The Botswana analysis and the WHO guidelines agreed on most recommendations related to selection and training, terms of service and programme implementation. This independent, but similar conclusion serves to reinforce the validity of the WHO guidelines. Even so, the Botswana analysis highlighted two areas not fully addressed by WHO’s CHW guidelines. 

First, a monitoring and evaluation framework for tracking CHWs is an important asset. Since 1978, WHO has asked Member States to report data on CHWs annually to the Global Health Observatory data repository.^13^ The repository houses over 1000 priority health indicators, including some pertaining to CHWs, and uses standard methods to make data comparable across countries. However, the definition of CHW is not standardized; WHO lets each country use its own definition. Moreover, the data repository is underutilized. Only 57 Member States have ever reported CHW numbers to the repository. Only six Member States have reported CHW numbers more than 10 times and the highest number of reports submitted in a year were from 22 Member States in 2004.^13^ Improving the indicators for CHWs and increasing use of the data repository could be an important step in implementing the WHO’s CHW guidelines. 

Second, a national policy for implementing the WHO’s CHW guidelines can be viewed as an indication of government commitment to include CHWs more formally in the health workforce. In Botswana, stakeholders felt strongly that a national policy would ensure an enabling and organized environment in which each CHW could contribute effectively to the desired results.

The Botswana analysis confirms that the WHO guidelines for CHWs are a useful tool for action at the national level. Countries like Botswana, which see CHWs as an extension of their primary health-care workforce, can adopt these guidelines to reach their service delivery goals. More robust and systematic monitoring of the WHO guideline will identify trends in evening-out imbalances in CHW integration in the health system, provide evidence for CHW contributions to health system performance and track WHO Member States’ commitment to universal health coverage.
